# Effect of Reduced-Dose Capecitabine Plus Cetuximab as Maintenance Therapy for *RAS* Wild-Type Metastatic Colorectal Cancer: A Phase 2 Clinical Trial

**DOI:** 10.1001/jamanetworkopen.2020.11036

**Published:** 2020-07-20

**Authors:** Lu Wang, Ying Liu, Xianli Yin, Weijia Fang, Jianping Xiong, Ben Zhao, Mingsheng Zhang, Yanmei Zou, Hong Qiu, Xianglin Yuan

**Affiliations:** 1 Department of Oncology, Tongji Hospital, Tongji Medical College, Huazhong University of Science and Technology, Wuhan, Hubei, China; 2 Department of Medical Oncology of Zhengzhou University Affiliated Cancer Hospital, Henan Cancer Hospital, Zhengzhou, Henan, China; 3 Gastroenterology and Urology Department, Hunan Cancer Hospital & The Affiliated Cancer Hospital of Xiangya School of Medicine, Central South University, Changsha, Hunan, China; 4 Department of Medical Oncology, The First Affiliated Hospital, School of Medicine, Zhejiang University, Hangzhou, Zhejiang, China; 5 Department of Oncology, The First Affiliated Hospital of Nanchang University, Nanchang, Jiangxi, China

## Abstract

**Importance:**

Fluorouracil-based chemotherapy combined with anti–epidermal growth factor receptor/vascular endothelial growth factor therapy is the standard first-line treatment for metastatic colorectal cancer followed by low-intensity maintenance therapy to balance the clinical efficacy and adverse effects (AEs). However, there have been concerns about the AEs of capecitabine plus cetuximab as a maintenance therapy in patients with *RAS* wild-type metastatic colorectal cancer.

**Objective:**

To evaluate the biological activity and safety of capecitabine plus cetuximab as a novel maintenance therapy for *RAS* wild-type metastatic colorectal cancer.

**Design, Setting, and Participants:**

This phase 2 prospective clinical trial was conducted from April 29, 2016, to April 29, 2019, at 5 centers in China. Patients diagnosed as having *RAS* wild-type metastatic colorectal cancer were recruited to receive fluorouracil-based cytotoxic agents combined with cetuximab followed by capecitabine plus cetuximab for maintenance therapy. Forty-seven patients with histologically confirmed metastatic colorectal cancer and genetic test results showing a wild-type *RAS* were enrolled in maintenance therapy.

**Interventions:**

Induction therapy for patients with *RAS* wild-type metastatic colorectal cancer was 8 to 12 cycles of fluorouracil-based chemotherapy combined with cetuximab. After stable disease status or better was achieved, reduced-dose capecitabine plus cetuximab was administered for maintenance therapy.

**Main Outcomes and Measures:**

The primary end point was progression-free survival during maintenance therapy. The secondary end points were total progression-free survival, overall survival, quality of life, safety, and toxic effects of treatment.

**Results:**

Forty-seven patients were enrolled in maintenance therapy, with a median age of 52 years (range, 25-81 years) and 32 (68%) of them being men. The median maintenance progression-free survival was 7.2 (95% CI, 5.8-8.6) months. The median progression-free survival was 12.7 (95% CI, 11.8-15.4) months. The median overall survival was 27.4 (95% CI, 21.4-35.5) months. Grade 3 to 4 AEs during induction therapy included neutropenia (4 patients [9%]), diarrhea (4 patients [9%]), nausea or vomiting (3 patients [6%]), rash acneiform (10 patients [21%]), and hand-foot syndrome (8 patients [17%]). Grade 3 to 4 AEs during maintenance therapy included diarrhea (2 patients [4%]), rash acneiform (8 patients [17%]), and hand-foot syndrome (5 patients [11%]).

**Conclusions and Relevance:**

Reduced-dose capecitabine plus cetuximab after initial chemotherapy is a novel maintenance therapy for patients with *RAS* wild-type metastatic colorectal cancer that achieved good outcomes and tolerable nonserious AEs.

**Trial Registration:**

ClinicalTrials.gov Identifier: NCT02717923

## Key Points

**Question:**

Does the combination of capecitabine and cetuximab as maintenance therapy achieve expected progression-free survival and overall survival among patients with *RAS* wild-type metastatic colorectal cancer?

**Findings:**

Forty-seven patients with *RAS* wild-type metastatic colorectal cancer were recruited in this phase 2 clinical trial. The median maintenance progression-free survival was 7.2 months, and the median overall survival was 27.4 months.

**Meaning:**

The combination of capecitabine and cetuximab as maintenance therapy achieved good outcomes and may be an alternative choice for patients with *RAS* wild-type metastatic colorectal cancer.

## Teaser

This phase 2 clinical trial evaluates the biological activity and safety of capecitabine plus cetuximab as a novel maintenance therapy for *RAS* wild-type metastatic colorectal cancer.

## Introduction

Colorectal cancer is the third most common and the second most lethal cancer in the United States.^1,2^ Fluorouracil-based chemotherapy (combined with oxaliplatin or irinotecan) plus anti–epidermal growth factor receptor/vascular endothelial growth factor (anti-EGFR/VEGF) therapy is the standard first-line treatment for metastatic colorectal cancer, with an overall median survival of 29 to 30 months.^3,4,5,6,7^ After stable disease status or better is achieved, switching to a low-intensity or low-toxicity maintenance therapy could balance the clinical efficacy and adverse effects (AEs).^8,9,10,11,12,13^

In the OPTIMOX1 and OPTIMOX2 trials,^8,9^ fluorouracil as maintenance therapy after leucovorin calcium and fluorouracil with oxaliplatin 4 (FOLFOX4) induction therapy vs continuous FOLFOX4 showed a similar effectiveness, fewer AEs, and better progression-free survival (PFS) and overall survival (OS) compared with an observation group. The results of those trials suggested that the use of a single cytotoxic agent could be valuable after intensive therapy. The MACRO, MACRO-2, and NORDIC-VII trials^11,13,14^ also concluded that single-agent bevacizumab or cetuximab maintenance therapy was more tolerable than continued induction therapy. In addition, the CAIRO3 trial^12^ found that bevacizumab combined with capecitabine for maintenance therapy obtained better results compared with an observation group, suggesting that the combination could achieve maximal clinical efficacy. Moreover, the MACBETH trial^15^ tested maintenance therapy with cetuximab or bevacizumab after induction therapy with chemotherapy combined with cetuximab. The results showed that the group treated with cetuximab achieved better clinical efficacy than the group treated with bevacizumab, although the difference was not statistically significant. For patients with wild-type *RAS* (OMIM 190070 for *KRAS* and 164790 for *NRAS*) or *BRAF* (OMIM 164757) metastatic colorectal cancer (mCRC), the findings of previous trials^14,16^ suggested that an EGFR-targeted drug may be the optimal maintenance therapy after induction chemotherapy in combination with the EGFR-targeted drug. Consistent with this notion, in the VALENTINO trial,^17^ maintenance therapy with the EGFR-targeted drug panitumumab combined with fluorouracil achieved higher PFS and OS than panitumumab alone. However, this regimen is not the most convenient strategy because fluorouracil is an injectable drug.

Therefore, oral administration of capecitabine combined with the EGFR-targeted drug cetuximab as maintenance therapy could be an alternative choice after induction chemotherapy combined with cetuximab for patients with *RAS* or *BRAF* wild-type mCRC. However, there have been concerns about the safety of this approach after studies^4,18,19^ reported AEs, including diarrhea, rash acneiform, and hand-foot syndrome. To evaluate the biological activity and safety of capecitabine plus cetuximab as a novel maintenance therapy for *RAS* wild-type mCRC, we performed a phase 2 prospective clinical trial. We aimed to test whether this maintenance therapy combination would result in expected outcomes, have only tolerable nonserious AEs, and provide evidence for future phase 3 clinical trials.

## Methods

### Study Design and Participants

This investigation was a multicenter, single-arm, phase 2 prospective clinical trial to evaluate the biological activity and safety of capecitabine plus cetuximab as a novel maintenance therapy after induction therapy consisting of fluorouracil-based chemotherapy combined with cetuximab. The trial protocol is available in the eAppendix in the Supplement. According to work by Simon,^20^ we planned to enroll at least 40 patients in maintenance therapy, and 24 patients (60%) should have been alive and meeting the primary end point (PFS during maintenance therapy [mPFS]). In the first stage, 23 patients were recruited, 18 (78%) of whom reached progression-free status in the ninth month of maintenance therapy. From April 29, 2016, to April 29, 2019, patients were recruited with histologically confirmed mCRC, genetic test results showing a wild-type *RAS*, age 18 years or older, and Eastern Cooperative Oncology Group performance status of 0 to 1. Before surgery, radiotherapy, liver resection, and other local treatments were permitted except for chemotherapy within 12 months. The incidence of AEs was assessed during maintenance therapy and the entire treatment.

The trial was approved by the ethics committee or institutional review board at each center. It was conducted according to the Declaration of Helsinki^21^ and with the Good Clinical Practice Guidelines of the International Conference on Harmonization.^22^ All patients provided written informed consent before study enrollment. This study followed the Strengthening the Reporting of Observational Studies in Epidemiology (STROBE) reporting guideline.

### Investigational Treatment

Investigational treatment began with a chemotherapy regimen of fluorouracil plus oxaliplatin or irinotecan combined with cetuximab in 8 to 12 cycles of induction therapy. After stable disease status or better was achieved, patients entered the maintenance therapy phase, receiving a combination of reduced-dose capecitabine (1000 mg/m^2^ orally twice a day on days 1-14 every 3 weeks) and cetuximab (400 mg/m^2^ orally on day 1 of the first week and then 250 mg/m^2^ on day 1 every week or 500 mg/m^2^ on day 1 every 2 weeks.). Treatment continued until disease progression, intolerable AEs, or death occurred.

### Outcomes

The primary end point of the study was mPFS with reduced-dose capecitabine plus cetuximab. The secondary end points were total PFS, OS, quality of life, safety, and toxicity of treatment.

### Study Assessments

To evaluate the response to metastatic lesions after treatment, magnetic resonance imaging or computed tomography was performed every 6 weeks until disease progression. Disease was assessed radiographically according to the Response Evaluation Criteria in Solid Tumors (RECIST), version 1.1.^23^ The mPFS was defined as the time from the beginning of maintenance therapy to first disease progression or death. The total OS was defined as the time from follow-up to withdrawal from the trial or death, and the OS from maintenance started at the time of the maintenance stage. The PFS was defined as the time from induction therapy to first disease progression or death. The quality of life (QOL) was assessed by QOL questionnaire (European Organization for Research and Treatment of Cancer Quality of Life Questionnaire–Core 30).^24^ Treatment-related AEs were graded according to the National Cancer Institute Common Terminology Criteria for Adverse Events (version 4.03).^25^

### Statistical Analysis

The biological activity and safety data were analyzed from April 29, 2016, to April 29, 2019. The Kaplan-Meier method was used to assess mPFS, PFS, and OS with 95% CIs and time-to-event data. Descriptive statistics were used for patient demographics and other characteristics. Biological activity analyses were evaluated in the intent-to-treat population receiving at least 1 cycle of maintenance therapy. Safety data described the rate of AEs with grade and the grade 3 to 4 AEs during maintenance therapy as well as the entire treatment for the intent-to-treat population. For the final analysis, the data cutoff date was December 15, 2019, with 47 patients enrolled in maintenance therapy assessed with survival curves. SAS (version 9.4; SAS Institute Inc) and GraphPad Prism 6 (GraphPad Software Inc) were used for statistical analysis and survival curves.

## Results

Sixty-one patients with *RAS* wild-type mCRC were enrolled in induction therapy from April 29, 2016, to April 29, 2019, at 5 centers in China. They received 8 to 12 cycles of fluorouracil-based chemotherapy combined with cetuximab. The patients entered into maintenance therapy after reaching stable disease status or better. Fourteen patients were excluded, including 9 patients with disease progression, 4 patients with intolerable AEs, and 1 patient who died.

**Figure 1.**
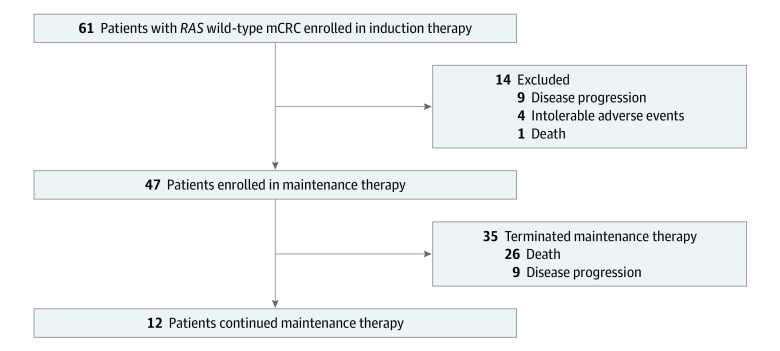
Patient Enrollment mCRC indicates metastatic colorectal cancer.

Forty-seven patients entered this phase 2 prospective clinical trial and were enrolled in maintenance therapy consisting of capecitabine plus cetuximab (Figure 1). The characteristics and disease status of patients are listed in Table 1. Their median age was 52 years (range, 25-81 years), and 32 (68%) of them were men.

**Table 1.** Characteristics and Disease Status in 47 Patients

Variable	No. (%)
Age, median (range), y	52 (25-81)
Male sex	32 (68)
Tumor location	
Rectum	25 (53)
Colon	22 (47)
Induction therapy	
Fluorouracil plus oxaliplatin	27 (57)
Fluorouracil plus irinotecan hydrochloride	20 (43)
Prior CRC treatment	
Surgery	13 (28)
Radiotherapy	3 (6)
Interventional therapy	5 (11)
6-mo Disease status	
Disease progression	14 (29)
Stable disease	21 (45)
Partial response	12 (26)

Abbreviation: CRC, colorectal cancer.

**Figure 2.**
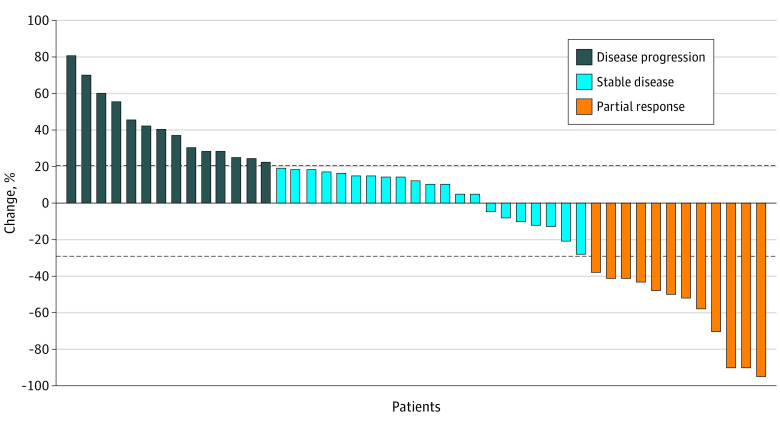
Six-Month Disease Status During Maintenance Therapy by Response Evaluation Criteria in Solid Tumors (RECIST), Version 1.1 The top and bottom dashed lines indicate the dividing line of stable disease. Each bar represents a patient.

**Figure 3.**
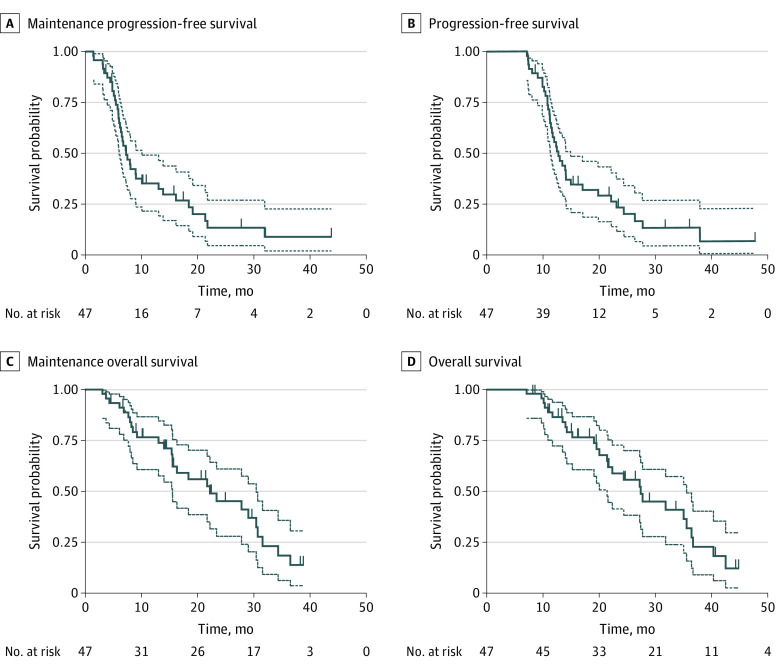
Kaplan-Meier Analysis of Survival Outcomes Dashed lines represent 95% CIs. Short vertical lines represent censored patients.

Among the 47 patients, the median follow-up was 30.4 (95% CI, 27.3-33.1) months. Fourteen patients (29%) had disease progression, 21 patients (45%) had stable disease, and 12 patients (26%) had partial response in the 6-month evaluation period (Table 1 and Figure 2). Disease progression survival assessment was performed for 47 patients: 12 patients continued maintenance treatment, 9 patients had second-line treatment, and 26 patients died. The median mPFS was 7.2 (95% CI, 5.8-8.6) months (Figure 3A). The median PFS was 12.7 (95% CI, 11.8-15.4) months (Figure 3B), with a 12-month PFS rate of 62% (95% CI, 46%-75%). The median maintenance OS was 22.2 (95% CI, 15.6-30.7) months (Figure 3C), with 21 of 47 patients (45%) still alive. The median OS was 27.4 (95% CI, 21.4-35.5) months (Figure 3D).

Quality of life was assessed in the 47 patients. Impaired emotional functioning, poor social relationships, fatigue, diarrhea, and treatment interference with daily activities were improved in maintenance therapy compared with induction therapy (eTable in the Supplement). The final average dosage of capecitabine was 900 mg/m^2^ to avoid AEs. Treatment-related toxicity and AEs were tolerable in the maintenance stage, with no AE-related deaths during the entire treatment. Grade 3 to 4 AEs were observed in 28% (13 of 47) of patients during induction therapy and in 21% (10 of 47) of patients during maintenance therapy. Grade 3 to 4 AEs during induction therapy included neutropenia (4 patients [9%]), diarrhea (4 patients [9%]), nausea or vomiting (3 patients [6%]), rash acneiform (10 patients [21%]), and hand-foot syndrome (8 patients [17%]). Grade 3 to 4 AEs during maintenance therapy included diarrhea (2 patients [4%]), rash acneiform (8 patients [17%]), and hand-foot syndrome (5 patients [11%]) (Table 2).

**Table 2.** Adverse Effects During Induction Therapy and Maintenance Therapy in 47 Patients by Grade

Variable	Induction therapy, No. (%)		Maintenance therapy, No. (%)	
	Grade <3	Grade ≥3	Grade <3	Grade ≥3
Neutropenia	27 (57)	4 (9)	2 (4)	0
Thrombocytopenia	10 (21)	0	3 (6)	0
Anemia	7 (15)	0	2 (4)	0
Diarrhea	19 (40)	4 (9)	15 (32)	2 (4)
Nausea or vomiting	18 (38)	3 (6)	2 (4)	0
Hepatotoxicity	9 (19)	0	4 (9)	0
Rash acneiform	30 (64)	10 (21)	28 (60)	8 (17)
Fatigue	19 (40)	0	10 (21)	0
Hand-foot syndrome	27 (57)	8 (17)	23 (49)	5 (11)
Fever	2 (4)	0	1 (2)	0

## Discussion

In patients with *RAS* or *BRAF* wild-type mCRC, clinical evidence is lacking on the use of a low-intensity or low-toxicity maintenance therapy after fluorouracil-based chemotherapy combined with anti-EGFR/VEGF therapy. This multicenter, single-arm, phase 2 prospective clinical trial enrolled 47 patients treated with a novel maintenance therapy that combined capecitabine plus cetuximab. The median mPFS was 7.2 months, and the median maintenance OS was 22.2 months; the median PFS was 12.7 months, and the median OS was 27.4 months. Grade 3 to 4 AEs were observed in 28% (13 of 47) of patients during induction therapy and in 21% (10 of 47) of patients during maintenance therapy. Maintenance therapy achieved the expected biological activity and tolerable AEs. From the pharmacokinetic point of view, no clinically relevant effect of cetuximab on capecitabine pharmacokinetic parameters and metabolic conversion could be detected. Therefore, the coadministration of capecitabine plus cetuximab seems to be safe, without altering the plasma concentration–time profiles.^26^

Our median PFS was 12.7 months, which was better than the 10.2 months achieved by the VALENTINO trial^17^ with the EGFR-targeted drug panitumumab combined with fluorouracil. The MACRO2 trial^13^ explored the efficacy of maintenance with either cetuximab only or chemotherapy plus cetuximab. For the cetuximab-only approach, the results showed a median PFS of 9 months and a median OS of 25 months, which was comparable to the modified FOLFOX6 (fluorouracil, leucovorin, and oxaliplatin) combined with cetuximab approach. The study confirmed that cetuximab alone can achieve a similar effect as 3 drugs plus cetuximab. Similarly, in the NORDIC-VII trial,^14^ maintenance therapy with cetuximab alone, with a median PFS of 7.5 months and a median OS of 21.4 months, was more clinically effective than FLOX (fluorouracil/leucovorin and oxalipatin) combined with cetuximab. Other trials that explored the effect of single agents, chemotherapy drugs, or their combination for maintenance therapy were OPTIMOX,^8,9,27^ CAIRO3,^12^ COIN-B,^28^ Nordic ACT2,^18^ MACRO,^11^ MACRO2,^13^ AOI0207,^29^ and MACBETH,^15^ as well as other studies.^15,27,29,30^ The results of the OPTIMOX1 trial^8^ suggested a stop-and-go strategy for mCRC treatment (eFigure in the Supplement). In that trial measuring the duration of disease control as the primary end point, disease control lasted 9.0 months for the FOLFOX7-treated group and 10.6 months for the maintenance group. In another phase 2 trial,^31^ 6 cycles of XELOX (capecitabine and oxaliplatin) induction therapy followed by capecitabine maintenance achieved a PFS of 8.0 months and an OS of 20.0 months. Compared with OPTIMOX1, the findings of the OPTIMOX2 trial^9^ suggested that patients can still benefit from fluorouracil-based maintenance therapy after intensive treatment. Therefore, according to that study, fluorouracil-based agents were the backbone for maintenance therapy. In the analysis of the maintenance efficacy, the duration of 2 stages (induction therapy and maintenance therapy) could influence the total PFS together. Longer fluorouracil-based induction therapy could increase PFS.

In the present trial, AEs and toxic effects were observed most often in maintenance therapy. The occurrence of grade 3 to 4 AEs during maintenance therapy was notably different from that in induction therapy. In the OPTIMOX1 trial,^8^ the maintenance group had a lower frequency of grade 3 to 4 AEs than the continuously treated group (48.2% vs 54.4%). Oxaliplatin-free maintenance after induction therapy achieved a similar clinical outcome. The response rate, PFS, and OS in our maintenance therapy could provide a theoretical basis for reducing treatment with oxaliplatin. In the OPTIMOX2 trial,^9^ the most common grade 3 to 4 AEs during maintenance therapy were neutrophilic granulosis (9.8%), neuropathy (4.9%), hand-foot syndrome (4.9%), mucositis (3.3%), and thrombocytopenia (1.6%). However, in the MACRO2 trial,^13^ neutropenia in the cetuximab group was greater than that in the cetuximab plus chemotherapy group, and deaths related to mucositis and AEs were the same. In the COIN trial,^10,32^ the use of capecitabine affected the occurrence of grade 3 to 4 AEs, including neutropenia, diarrhea, nausea or vomiting, and peripheral neuropathy. For combined treatment with capecitabine plus cetuximab, diarrhea, rash acneiform, and hand-foot syndrome may be the first-appearing AEs.

The MACRO2 trial^13^ reported differences between cetuximab only vs modified FOLFOX6 combined with cetuximab in the frequency of grade 3 to 4 AEs (15% vs 24% for rash acneiform and 7% vs 8% diarrhea). The use of capecitabine after XELOX induction therapy^31^ resulted in grade 3 to 4 AEs, including diarrhea (2.4%) and hand-foot syndrome (2.4%). In a trial^30^ administering CAPIRI (capecitabine and irinotecan) vs CAPOX (capecitabine and oxaliplatin) combined with cetuximab, rash acneiform was observed in 12.4% vs 20.5%, hand-foot syndrome in 12.4% vs 22.7%, and diarrhea in 15.7% vs 9.3%. In our trial, it should be noted that the final dosage of capecitabine was 900 mg/m^2^ to avoid AEs. The maintenance therapy in our study was safer than CAPIRI vs CAPOX combined with cetuximab, and the incidence of rash acneiform in maintenance therapy was lower than that in induction therapy. During maintenance therapy, we observed grade 3 to 4 rash acneiform in 8 patients (17%), hand-foot syndrome in 5 patients (11%), and diarrhea in 2 patients (4%). It should be pointed out that 47 patients (60%) had grade 1 to 2 skin toxic effects, and more AEs were observed in our trial compared with previous research in patients treated with cetuximab only.^13^ However, no patients experienced serious AEs in the present study.

### Limitations

A limitation of this multicenter, single-arm, phase 2 prospective clinical trial is that control groups were lacking. A future phase 3 clinical trial^33^ will compare cetuximab plus capecitabine vs cetuximab alone as a control group.

## Conclusions

In patients with *RAS* wild-type mCRC, good outcomes and tolerable nonserious AEs can be achieved with the combination of capecitabine plus cetuximab as maintenance therapy after induction therapy consisting of fluorouracil-based chemotherapy combined with cetuximab. A future phase 3 clinical trial^33^ will compare cetuximab alone vs capecitabine alone as control groups.

## Notes

Supplement. **eAppendix.** Trial Protocol of the Cohort Study **eTable.** Quality of Life in Induction Treatment and Maintenance Treatment **eFigure.** Progression-Free Survival Time of Different Maintenance Treatment Strategies
