# Supplementary material for: Effect of Reduced-Dose Capecitabine Plus Cetuximab as Maintenance Therapy for RAS Wild-Type Metastatic Colorectal Cancer: A Phase 2 Clinical Trial
Source: JAMA Netw Open. 2020 Jul 20;3(7):e2011036. doi: 10.1001/jamanetworkopen.2020.11036 (PMC7372324; doi:10.1001/jamanetworkopen.2020.11036)
Supplement: Supplement. — eAppendix. Trial Protocol of the Cohort Study eTable. Quality of Life in Induction Treatment and Maintenance Treatment eFigure. Progression-Free Survival Time of Different Maintenance Treatment Strategies [file jamanetwopen-3-e2011036-s001.pdf]

Supplementary Online Content

Wang L, Liu Y, Yin X, et al. Effect of reduced-dose capecitabine plus cetuximab as maintenance therapy for *RAS* wild-type metastatic colorectal cancer: a phase 2 clinical trial. *JAMA Netw Open*. 2020;3(7):e2011036. doi:10.1001/jamanetworkopen.2020.11036

eAppendix. Trial Protocol of the Cohort Study

eTable. Quality of Life in Induction Treatment and Maintenance Treatment

eFigure. Progression-Free Survival Time of Different Maintenance Treatment Strategies

This supplementary material has been provided by the authors to give readers additional information about their work.

© 2020 Wang L et al. *JAMA Network Open*.

eAppendix. Trial Protocol of the Cohort Study

Protocol

Study Design and Participants

This was a multi-center, single-arm, phase 2 clinical trial to evaluate the biological activity and safety of capecitabine plus cetuximab after an induction chemotherapy based of 5-FU with cetuximab. The study protocol in detail was provided (eMethods 1). According to Simon two-stage designs in 2-side (α=0.05, β=0.80), we planned to enroll at least 40 patients in final maintenance treatment and 24 patients (60%) should have been alive and have met the primary endpoint. In the primary stage, 23 patients were recruited, of which 18 patients (78%) more than 60% reached a progression free status in the ninth month. Patients were recruited from April 2016 to April 2019 with histologically confirmed mCRC, genetic test showing a wild type *RAS*, 18 years of age or more, Eastern Cooperative Oncology Group performance status of 0-1. Prior to surgery, radiotherapy, liver resection and other local treatment were permitted except for chemotherapy within 12 month.

The incidence of AEs was assessed during maintenance and entire treatment. The study was approved by the ethics committee or institutional review board at each center. It was conducted according to the Declaration of Helsinki and with the Good Clinical Practice Guidelines of the International Conference on Harmonization. The report followed STROBE reporting guideline for the cohort study and all patients provided written informed consent before the enrollment.

Investigational Treatment

We started with chemotherapy regimen of 5-FU plus oxaliplatin or irinotecan combined with cetuximab in 8-12 cycles of induction therapy. After reaching a stable disease status or better, patients entered the maintenance phase, receiving a combination of capecitabine (1000mg/m^2 orally, twice a day on days 1-14 every 3 weeks) and cetuximab (400mg /m^2 on day 1 of the first week, then 250 mg/m^2 on day 1 every week or 500 mg/m^2 on day 1 every 2 weeks.). Treatment would continue until

© 2020 Wang L et al. *JAMA Network Open*.

the disease progressed, death or unacceptable AEs occurred.

Outcomes

The primary outcome of the study was the maintenance progression-free survival (mPFS) during the capecitabine and cetuximab treatment. The secondary outcomes included the total PFS, the OS from maintenance, the total OS and 6-month overall response rate. Quality of life (QOL) and treatment related toxicity were analyzed and graded to evaluate the safety .

Study Assessments

In order to evaluate the response to metastatic lesions after treatment, magnetic resonance imaging or computed tomography were performed every 6 weeks until the disease progression. The disease was assessed radiographically according to the Response Evaluation Criteria in Solid Tumors (RECIST, version 1.1)[21]. mPFS was defined as the interval of time from the beginning of maintenance treatment to the first disease progression or death. The total OS was the interval of time from the follow-up to death or withdrawal and the OS from maintenance started at the time of the maintenance stage. The PFS was defined as the interval of time from the induction treatment to the first disease progression or death. QOL was assessed by QOL questionnaire (European Organization for Research and Treatment of Cancer [EORTC] Quality of Life Questionnaire-Core 30 [QLQ-C30]). Treatment-related AEs were graded according to the National Cancer Institute Common Terminology Criteria for Adverse Events, version 4.03.

© 2020 Wang L et al. *JAMA Network Open*.

Statistical Analysis

Safety and biological activity data were analyzed from April 2016 to April 2019. The Kaplan-Meier approach was performed for the distribution of mPFS, PFS, OS with 95% confidence interval and time-to-event data. Descriptive statistics was used for demographic and basic characteristic of patients. Biological activity analyses were evaluated in the intention-to-treat (ITT) population receiving at least one cycle of maintenance treatment. Safety data described the adverse events rate with grade and 3-4 grade AEs in the whole treatment and maintenance treatment for the ITT population. In the final analysis, the data cut off was in December 15, 2019, with a total of 47 patients were enrolled in the maintenance therapy assessed for survival curves. SAS (SAS Institute Inc), version 9.4 and GraphPad Prism 6 were used for statistical analysis and survival curve.

© 2020 Wang L et al. *JAMA Network Open*.

eTable. Quality of Life in Induction Treatment and Maintenance Treatment

	**Induction treatment vs. baseline**		**Maintenance treatment vs. induction treatment**	
	OR (95% CI)	*P* value	OR (95% CI)	*P* value
**Functional scales**				
Impaired physical functioning	1.03 (0.94-1.08)	0.14	0.93 (0.87-1.06)	0.21
Impaired role functioning	1.04 (0.72-1.14)	0.38	0.85 (0.81-1.10)	0.37
Impaired emotional functioning	1.07 (0.82-1.18)	0.47	0.82 (0.72-0.94)	0.028
Impaired cognitive functioning	1.12 (0.97-1.21)	0.51	0.91 (0.79-1.03)	0.28
Impaired social functioning	1.09 (0.92-1.17)	0.32	0.78 (0.61-0.86)	0.013
**Symptom scales**				
Fatigue	1.17 (0.97-1.24)	0.46	0.71 (0.53-0.92)	0.041
Nausea or vomiting	1.05 (0.87-1.16)	0.71	0.87 (0.81-1.03)	0.47
Pain	1.02 (0.81-1.23)	0.88	0.98 (0.90-1.03)	0.65
Dyspnoea	1.12 (0.96-1.16)	0.53	0.84 (0.80-0.97)	0.22

© 2020 Wang L et al. *JAMA Network Open*.

Insomnia	1.06 (0.91-1.18)	0.34	0.92 (0.82-1.02)	0.27
Appetite loss	1.09 (0.97-1.20)	0.74	1.02 (0.81-1.13)	0.67
Constipation	1.12 (0.90-1.19)	0.49	1.12 (0.91-1.17)	0.35
Diarrhoea	1.32 (1.16-1.43)	0.032	0.67 (0.56-0.78)	0.026
Dry or score mouth	1.02 (0.83-1.19)	0.13	0.87 (0.79-1.16)	0.29
Problems eating or drinking	1.13 (0.88-1.17)	0.61	0.97 (0.92-1.06)	0.13
Problems handing small objects	1.08 (0.87-1.06)	0.53	0.94 (0.89-1.03)	0.32
Treatment interferes with daily activities	1.05 (0.91-1.17)	0.44	0.89 (0.69-0.97)	0.037
Treatment felt to have been worthwhile	0.9 (0.84-1.12)	0.15	0. 81 (0.75-1.02)	0.73
**Global scales**				
Global quality of life	1.09 (0.92-1.14)	0.31	0.89 (0.82-1.14)	0.47

© 2020 Wang L et al. *JAMA Network Open*.

eFigure. Progression-Free Survival Time of Different Maintenance Treatment Strategies

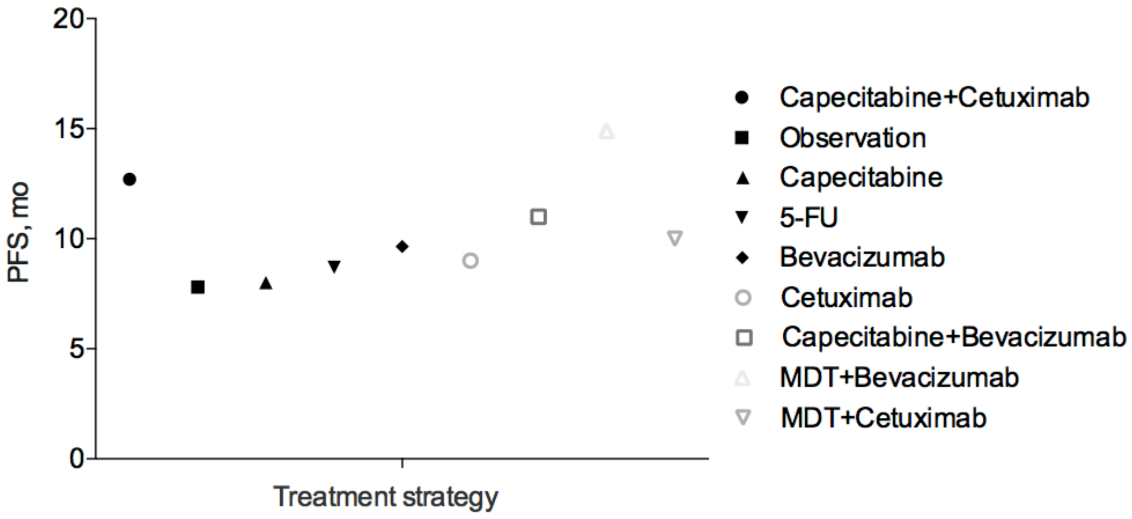

© 2020 Wang L et al. *JAMA Network Open*.
